# Impact of Three Strengthening Exercises on Dynamic Knee Valgus and Balance with Poor Knee Control among Young Football Players: A Randomized Controlled Trial

**DOI:** 10.3390/healthcare9050558

**Published:** 2021-05-10

**Authors:** Bartosz Wilczyński, Piotr Wąż, Katarzyna Zorena

**Affiliations:** 1 Department of Immunobiology and Environment Microbiology, Medical University of Gdańsk, 80-211 Gdańsk, Poland; katarzyna.zorena@gumed.edu.pl; 2 Department of Nuclear Medicine, Medical University of Gdańsk, 80-211 Gdańsk, Poland; piotr.waz@gumed.edu.pl

**Keywords:** knee kinetics, injury prevention, medial knee displacement, single leg squat, Y-Balance test

## Abstract

The observed dynamic knee valgus and the limited dynamic balance described in the literature are modifiable risk factors for injuries in athletes. Therefore, identification and appropriate prevention are crucial in managing the development of young athletes. The aim of the study was to assess the effectiveness of three exercises strengthening the muscles: gluteal medius, popliteal and tibialis posterior to reduce dynamic knee valgus and improve the dynamic balance of the lower limbs in young football players with poor knee control. A total of 134 footballers were assessed for eligibility, and finally 45 participants (age 12–15) met the inclusion criteria. Participants were assessed with 2D video kinematic analysis during single-leg squats to assess the knee valgus angles and the dynamic balance (Y-Balance Test). No significant interactions between groups (Control and Exercise) and time (baseline and after 6 week) were noted for dynamic valgus for the left and right knee (*p* > 0.05). For the dynamic balance, there were statistically significant results, but not clinically relevant for anterior, posteromedial, and composite direction for the right lower limbs and for the anterior direction for left lower limbs in the exercise group. However, there were no significant differences (*p* > 0.05) in all of the YBT scores for both lower limbs between groups. This study demonstrated that there were no statistically significant differences in dynamic knee valgus angles and dynamic balance values after 6 weeks of exercise program in young footballers with poor knee control. Future randomized trials should focus on more comprehensive exercises, where possible using biofeedback methods to improve knee kinematics.

## 1. Introduction

One of the most popular sports in the world is undoubtedly football, both among fans and active participants. Over 50% of registered footballers worldwide are 18 and under [1]. Milanovic et al. in 2015 showed that there are ~500 million active footballers in the world, including 300 million registered with football clubs [2]. The popularity of football among the child and adolescent population has many positive health effects [3]. On the other hand, training and playing soccer can contribute to sports injuries [4,5].

Injuries in children and youth football mainly affect the lower extremities [6]. The most common types are strains (muscle-tendon), contusions, and sprains (joint-ligament). The injury rate increases linearly in the range of 9 to 15 years among male footballers with a pronounced increase at age 13 [5,7]. Therefore, the prevention of injuries in that population of children and adolescents practicing sports is a key aspect of medical practice. The growing number of both overload and acute injuries in youth sports forces the need to look for new and more effective preventive programs. Known in the literature, programs such as FIFA 11+ and Prevention Exercise Program (PEP) appear to be effective in reducing the number of injuries overall [8,9,10]. However, serious injuries such as an anterior cruciate ligament ACL rupture in noncontact situations still occur, despite the use of preventive programs [8]. Moreover, studies have shown a worrying rise in the number of ACL injuries among children and adolescents in recent years [11,12]. Severe injuries have serious consequences on the physical and mental development of young athletes [4]. Therefore, there is a need to look for new targeted solutions, also as an addition to the existing preventive programs. Targeted preventive training may focus on a specific risk factor. One of the modifiable injury risk factors is neuromuscular control which includes dynamic knee valgus (DKV) and reduced dynamic balance [7,13]. The movement pattern consisting of the simultaneous movements of adduction and internal rotation of the hip, abduction of the knee, external rotation of the tibia, and eversion of the ankle joint with pronation of the feet is called the dynamic valgus knee. This pattern is treated as a risk factor for injuries such as ACL trauma and the occurrence of PFP [14,15,16,17]. Moreover, recent studies confirmed during video analysis that athletes suffering from ACL injuries landed in the position of dynamic knee valgus [18,19]. Moreover, knee valgus may influence the occurrence of patellofemoral pain (PFP) through the patella maltracking mechanism and increase the load on the patellofemoral joint [20]. Furthermore, young athletes who had increased valgus angles in frontal projection plane angle (FPPA) during Single Leg Squat (SLS) are associated with a risk of acute ankle injuries and are 2.7 times more likely to suffer from severe lower limb injuries [21]. A 3D analysis is a gold standard for the kinematic evaluation of DKV. However, an acceptable, cheaper, and easier to use method is a 2D analysis focusing on the assessment of the FPPA of the knee [22,23]. Moreover, researchers reported reliability, reproducibility, and good agreement in an average mean difference in FPPA between 2D and 3D analysis. In [22,24] The DKV pattern is observed in research in tests such as squat, landing, cutting, on the double-leg, and single-leg test. However, due to the evidence that ACL injuries mainly occur during single-leg tasks, we used Single Leg Squat (SLS) in our study [18,19].

Another risk factor for athlete’s lower limb injuries is decreased dynamic balance. The Y-Balance Test is a popular and reliable test used among athletes to assess dynamic balance. A decreased YBT score is associated with the occurrence of lower limb injuries in various populations [25,26,27]. Moreover, reduced neuromuscular efficiency, such as lower limb strength, may affect the control of dynamic balance [28].

Existing exercise programs to reduce knee valgus are mainly based on a large number of exercises at various stages (warm-up, plyometrics, strength), visual biofeedback exercises (for example, monitor or mirror), and balance training [29,30,31,32]. Proposed by Attwood et al. The 2018 Injury Prevention Exercise Program showed a 40% reduction in the incidence of lower limb injuries compared to the control group. The program lasted 42 weeks and consisted of a large number of balance exercises, perturbation and strengthening exercises, cutting and landing exercises that also required feedback [33].

The problems associated with preventive exercises include, among others: long execution time, specialist equipment required, difficulties in performing exercises requiring constant care of trainers or physiotherapists. The solution to the above problems is to use fewer exercises, shorten the execution time, choose less complicated exercises without the use of equipment, which can be carried out independently by children and adolescents in all conditions.

Our assumption was to provide a simple intervention, not time-consuming and possible to perform without specialized equipment also as home-exercises. Exercise programs are the most beneficial for people with low movement quality and with a risk of injury, we decided to include young athletes with poor knee control—DKV [34]. The cause of excessive knee valgus can be a combination of the muscular imbalance between hip abductors and adductors, external and internal rotators, and foot pronators and supinators [34,35,36,37]. We assumed that players with observed dynamic knee valgus may have a muscular imbalance predisposing to hip addiction, external tibia rotation and foot pronation in single-leg movement tasks. We hypothesized that muscles responsible for moving in the opposite direction to those predisposing to DKV should be strengthened to compensate for the knee’s axial alignment. Much attention was paid to the improvement of strength and activation of the hip and its role in controlling DKV. The weakness of hip abductors reduces the stability of the hip after loading it with single leg tasks, causing the inability to maintain a neutral hip and knee position. The findings of study Naematallah et al. shows that DKV during SLS occurred due to reduced coactivation of the gluteal muscles relative to the hip adductors. Therefore, injury prevention programs should include exercises to increase the activity of hip abductors [37]. Gluteus medius is the strongest hip abductor is most often described in the context of DKV [38]. Side-lying hip abduction exercise is classified as high-level activation of the gluteus medius [39]. Bell et al., 2013 recommend interventions to improve knee valgus using ankle exercises such as the tibialis posterior [34] This muscle performs the functions of foot adductor, invertor, and plantarflexor. Selective and effective activation is achieved by resisted foot adduction [40]. The popliteus muscle as an internal rotator of the shin during the knee acts as a dynamic stabilizer to control subtle movements in FPPA, supporting balance in single leg tasks. Due to the function and anatomy of this muscle, Nyland et al., 2008 proposed non-bearing exercise to improve activation of popliteus and effect on lower extremity injury preventions, also by reducing knee valgus and increasing knee stabilization [41,42].

The aim of our study was to evaluate the efficacy of the three strengthening exercises to reduce knee valgus angles on FPPA in SLS and improve dynamic balance in YBT in young football players. Our hypothesis was assumed that our simple exercise programme improves these modifiable risk factors.

## 2. Materials and Methods

### 2.1. Participants

**Figure 1:**
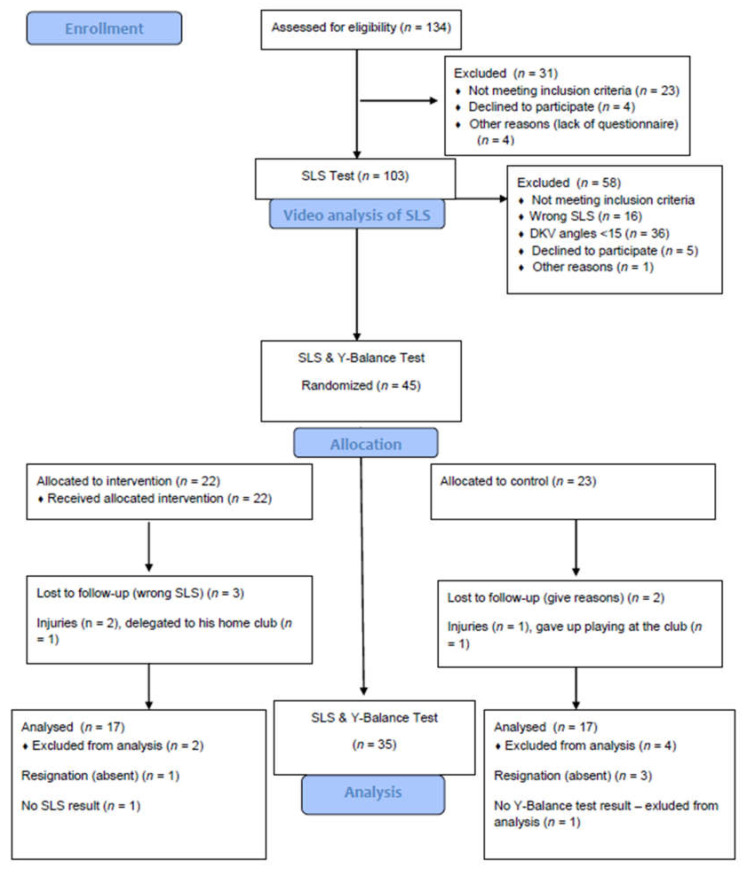
CONSORT flow diagram of recruitment and retainment.

Participants were recruited for the study from October to December 2018 from a local soccer club through invitations, brochures, and e-mails sent to the club’s coaches and administrative staff. 134 football players were assessed for eligibility. Ultimately, forty-five footballers met the inclusion criteria and took part in the study, All drop-outs are shown in Figure 1.

Young male amateur football players with poor knee control were qualified if they met the inclusion criteria: (1) written consent of the guardian/parent to participate in study (2) healthy, not reporting pain (3) consent to participate in sports competitions by a sports medicine physician (4) minimum 2 years of experience in training and football games, and (5) meeting the criterion of dynamic knee valgus (FPPA angle > 15° during the SLS test in 2D video analysis). Participants were initially screened by lead experienced physiotherapists and were classified as “poor knee control” in SLS test with >15 degrees of FPPA (knee moving into a valgus position) in 2D video analysis. This screening tool has been recommended and validated with 3D analysis for the presence of DKV [43,44] Participants were excluded from the study if (1) were unable to perform strengthening exercises and were unable to participate in sports activities due to health problems, (2) had undergone lower limb or torso surgery in the past 2 years. 

### 2.2. Study Design

A parallel-group randomized trial was used to compare the exercise group performing three strengthening exercises versus a control group without intervention. Participants were randomly assigned to the Exercise Group or Control Group in a blinded fashion following the first testing with a 1:1 ratio using a computer-based method (Figure 1). The Exercise Group was assigned to the 6-week exercise program. The control group did not perform exercises. Both groups participated without interruption in their sports activities. The trial has been designed and reported in accordance with the CONSORT guidelines [45]. Blinding of the participants and authors was not possible. The study was approved by the Independent Bioethical Committee for Scientific Research at the Gdańsk Medical University.

### 2.3. The Tests and Exercise Supervision among Young Football Players

All tests and exercise supervision took place in the sports hall at the Rehabilitation and Training Center in the afternoon (15–18) from December 2018 to May 2019. Body composition analysis was used by the InBody 270 analyzer (InBody Co., Seoul, Korea) and height measurment was made by the laser method (InKids, InBody Co., Seoul, Korea). 

#### 2.3.1. Single Leg Squat

The Single Leg Squat test was carried out in accordance with the methodology described in previous studies [24,46]. Before starting the test, participants watched the instructional video on the projector and have obtained verbal information. The researchers demonstrated correct squat performance from the lateral perspective so that they did not suggest the knee’s projection from the frontal plane. Markers in the form of the square kinesiotape patches were affixed in the anatomical points at the upper anterior iliac spine, at the middle point of the patella, and to the point of the ankle between the lateral and medial bones [24,47]. Participants were instructed to stand barefoot on a line formed of two white patches to standardize the test site. Then, during the verbal signal of the researcher, participant (1) bent his untested limb to an angle approximately 90°, (2) straightened the hip and knee of the tested limb, (3) stacked crossed the hands on the shoulders, (4) facing his eyes forward. Another signal from the researcher suggested starting the test. The maximum duration of the squat was set as 5 s measured by the rater [24]. Participants received information about the depth of the squat which was limited to approximately 60°. Participants after the instructions were able to practice the test (approximately four times) until they were ready to start. If the subject shifted his foot or heel, touched the ground with the free limb, lost balance, the test was considered unsuccessful and was repeated. Three attempts were made for the left and right lower limbs with a two-minute recovery between squats. The mean of the three trials was analyzed.

#### 2.3.2. Frontal Plane Projection Angle

FPPA was used to assess dynamic knee valgus angles. Two digital video cameras (GoProHero 4, GoPro, Inc., San Mateo, CA, USA) were located on tripods, the first at a distance of 2 m laterally and the second at the front 1 m at the knee joint level of the subject [24,47]. Free available traffic tracking software was used with the Kinovea^®^ (beta-version 0.8.26, Bordeaux, France) previously used in studies [22,48]. Kinovea has been demonstrated in the literature as a validated and reliable method for assessing angles and distances [49]. Markers adhered to the previously mentioned anatomical areas of the participants were points determining the alignment of the lower limbs. During video analysis in the frontal plane projection, the researcher calculated FPPA by measuring the angle marked by lines of the center of the patella, followed by the upper anterior iliac spine and a middle point between the lateral and medial ankle malleolus. The sagittal projection was analyzed to determine the minimum angle of knee bending (at least 60° for a successful sample) and included anatomical points: greater trochanter, lateral condyle of the tibia, and lateral malleolus. The starting angle of the test limb was determined at the moment of the single leg stance, and the maximum angle of the knee valgus was marked during the squat at the maximum deviation from the starting position (Figure 1). If during the video analysis, the researcher noticed the irregularities in the test performance, classified it as an unsuccessful attempt, without the possibility of repetition. The average of the three attempts from the FPPA value was used for analysis. 2D analysis of the frontal plane projection angle of the lower limb alignment was demonstrated a good correlation with 3D methods. Research has shown between-session Standard Error of Measurement (SEM) values of the 4° [24].

#### 2.3.3. Y-Balance Test

Y-Balance Test was used to evaluate the dynamic balance of the lower limbs. The test consists of the Y-Balance Test kit (Move2Perform, Evansville, IN, USA). Participants were instructed to keep the standing position one-legged with the hands placed at the hips on the leg placed on the stand arranged so that the big finger touches the horizontal line [50]. With a free limb, the participant moves the block towards the anterior (ANT), posterolateral (PL) and posteromedial (PM) directions, trying to get the most distant point, and then return to the starting position [51,52]. The study was carried out with recommendations for standardization of the protocol according to Plisky et al., 2008, (participants performed the test barefoot, six practice attempts for all directions to minimize the learning effect, video instruction on how to perform the test. Participants changed limbs for each direction alternately, the starting point of the laden load in the same position, permitted foot movements, i.e., heel or forefoot separation, any body movement is allowed, normalization to the length of the lower limb, same platform height) [53].

The length of the lower limbs was measured on the therapy table. The researcher straightened his legs to align the pelvis. The length was measured from the upper anterior iliac spine to the medial malleolus of the lower limb.

The subjects performed three repetitions of the left and right lower limbs in all directions. The test was considered unsuccessful at the moment when the person lost balance, did not return to the starting position, bounced off the platform or lost contact within an uncontrolled manner, the loaded foot moved to the horizontal line. The distance of the offset platform was registered as the result of a given limb for a specific direction with an accuracy of 1 cm. The result was normalized with the length of the limbs and described as a percentage (LL%) [51]. Composite (COM) reach distance for each lower limb was calculated as the sum of three directions divided by three times the length of the lower limbs and multiplied by 100 [54]. The mean of the three attempts in each direction for the left and right leg was used for the analysis. YBT showed good interrater and intrarater reliability in previous studies [53,54].

### 2.4. Exercise Program

**Figure 2:**
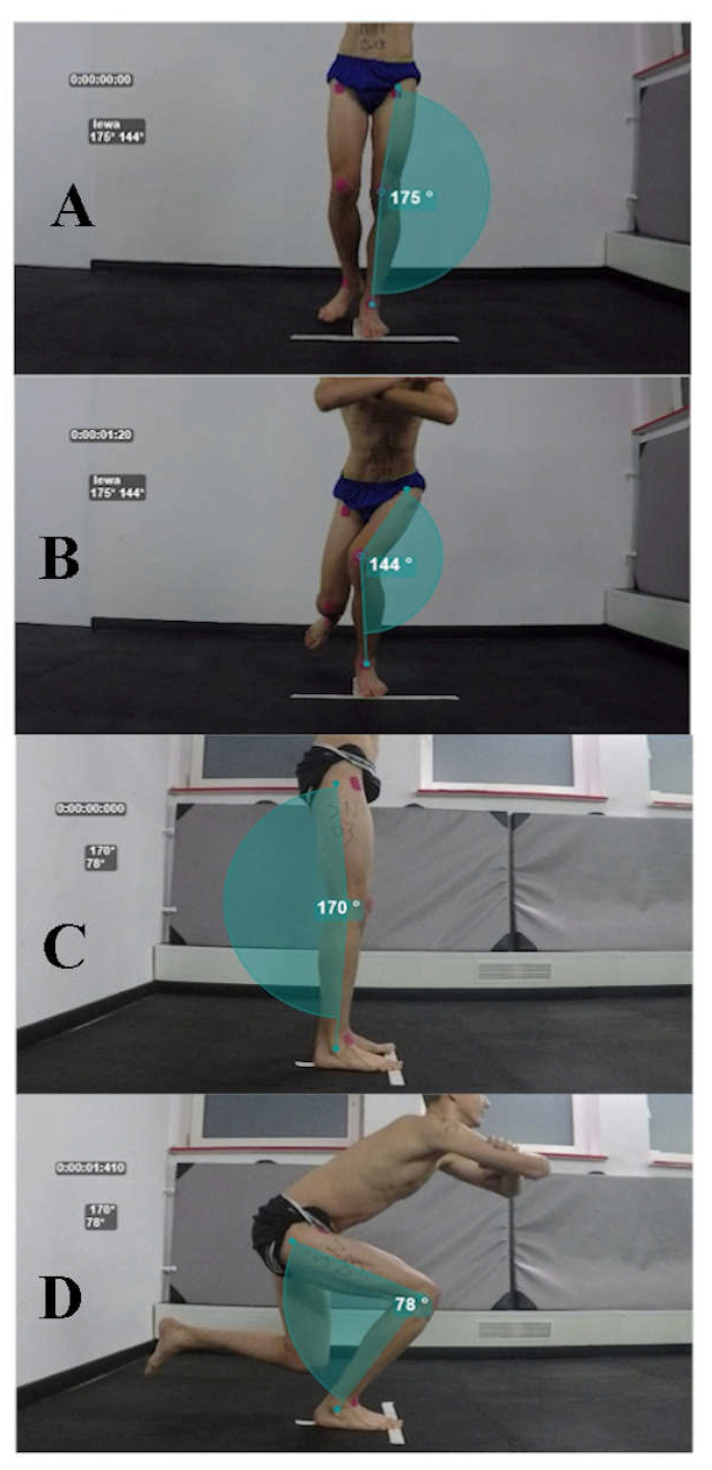
Video analyses of SLS test in the frontal and sagittal projection. (**A**,**C**)—starting angle, start of the test, (**B**,**D**)—maximum DKV, end of the test.

The exercise group was allocated to a 6-week training program, which took place three times per week independently at home and once per training under the control of physiotherapists and sports trainers. The program consisted of three muscle exercises using elastic rehabilitation band as external resistance. Exercises strengthening of the gluteus medius, popliteal and tibialis posterior muscles (Figure 2 and Table 1). These muscles affect the movements in the hip, knee and ankle joints, respectively. These exercises have been proposed by researchers in publications in the past [39,40,42]. Execution of muscle strengthening is based on the guidelines of the National Health of Sports Medicine on a combination of concentric contractions—1 s, isometric—2 s and eccentric—4 s to activate the muscle in the best possible way [55]. The interval between each set was 60 s, and the time between exercises was from 1 min to 3 min. Exercises were performed for the left and right lower limbs, respectively. The description and progression of these exercises show Table 1.

The elastic band was selected to the age of the participant. Younger participants aged 11–13 received a blue band with a thickness of 0.50 mm (weaker tension), and participants aged 14–16 received a black band with higher resistance of 0.65 mm (stronger tension).

Besides, participants received a package with information for the guardian/parent regarding motivational support and involvement in the exercises of the young athlete, illustrations with a detailed description of the exercises, a card with a specially generated website address to the base in which instructional videos about the exercise were posted and the method of tying the band.

The package also included a training diary with information on the progression of the weekly program. The task of the respondents was to mark the training days in the training diary as a verification of the exercise of home exercises.

### 2.5. Statistical Methods

The normality of the data was determined using the Shapiro–Wilk test. The results that met the criteria of normal distribution were presented as means ± SD. If the results were nonparametric then they were described as medians and interquartile range (IQR). Inter-group and intra-group comparisons are shown as means and standard deviations (SD). Comparisons between pretraining and post-training for non-parametrical results in each group were performed with the Wilcoxon signed-rank test, and both groups were compared using the Mann–Whitney U test. Comparisons between pretraining and post-training by the independent t-test were performed for parametric results. To compare differences between the pre and post-test values for the experimental and control groups a 2 × 2 (group × time) repeated measures ANOVA was used. The effect size was calculated as Cohen’s d with criteria for d values: ≤0.2 was considered small, ≤0.5 medium, and ≥0.8 large. A significance level of *p* ≤ 0.05 was set for all statistical analyzes. All data were processed with the Statistica software (Statistica 12).

## 3. Results

**Figure 3:**
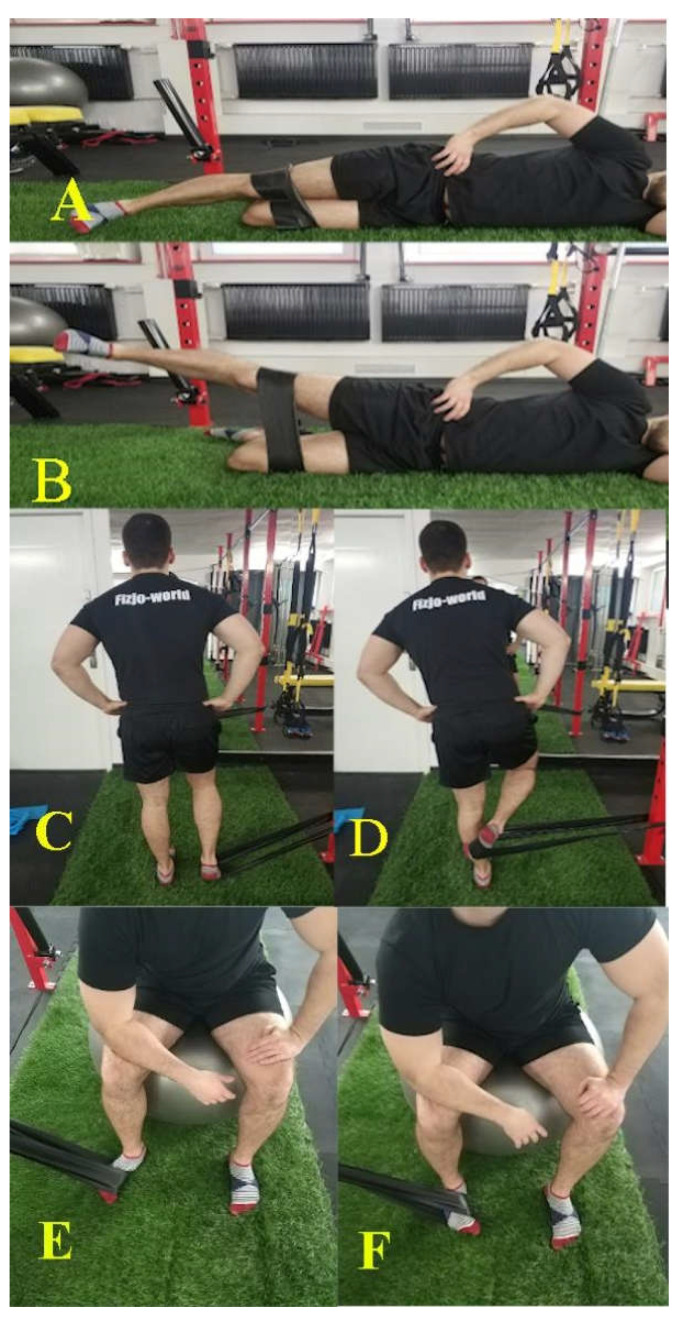
Initial and final exercise position. (**A**,**B**)—exercise of gluteus medium, (**C**,**D**)—exercise of politeus, (**E**,**F**)—exercise of posterior tibalis.

A total of 134 young male soccer academy players were screened from December 2018 to March 2019, 103 of which were invited for eligibility assessment. Ultimately 45 participants met the inclusion criteria and 58 were excluded (Figure 3). The groups did not differ from each other in the anthropometric characteristics and primary and secondary outcome (Table 2). In total, eight (24%) participants were lost at follow up, six in the control group, five in the intervention group, including three people due to an injury.

### 3.1. Primary Outcome

**Figure 4:**
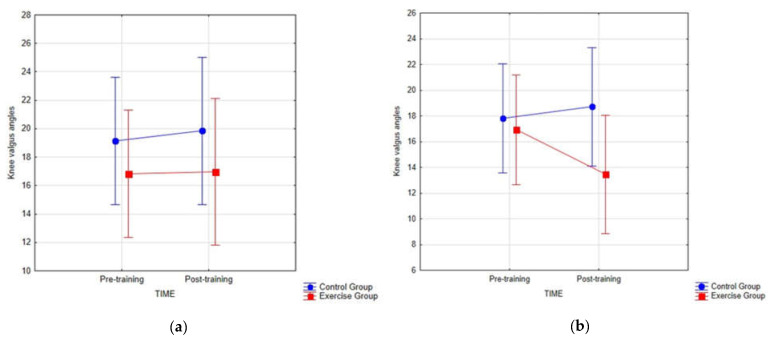
Mean with SD (°) for both groups at Pre-training and Post-training for the (**a**) right knee valgus, and (**b**) left knee valgus angles.

There were no significant interactions between the groups and the time in the dynamic knee valgus for the left (*p*-value = 0.291) and the right knee (*p*-value = 0.905; Figure 4) in the young male football players.

In the exercise group, the knee valgus angle values showed a tendency to decrease after the exercise program for left knee valgus (−2.62°) However, despite this difference, it was not statistically significant (*p*-value = 0.309) and was lower than SEM (defined in previous studies at 4°) [24].

The comparison between the exercise group and the control group showed no statistically significant changes in pretraining for the left (*p*-value = 0.426) and right knee (*p*-value = 0.469) and in post training for the left (*p*-value = 0.109) and right knee (*p*-value = 0.427). Pre- and post-training dynamic knee valgus values for the left and right knee are presented in Table 3.

### 3.2. Secondary Outcome

There were no statistic differences (*p* > 0.05) in all of the YBT scores for the right and for the left lower limbs between groups at pretraining and post-training.

In the exercise group, the YBT anterior score increased from (69.1; IQR = 8.1) at pretraining to (73.6; IQR = 8.3; *p*-value = 0.038) at post-training for right lower limbs, and from (68.3; IQR = 7.2) to (71.9; IQR = 7.3; *p*-value = 0.039) for the left lower limbs. The YBT posteromedial for the right lower limbs increased from pretraining (104.3; IQR = 12.3) to post training (110.0; IQR = 11.4; *p* = 0.006). The YBT composite for the right lower limbs increased from pretraining (92.2; IQR = 5.9) to post-training (97.9; IQR = 6.3; *p*-value = 0.014) among football players in the exercise group. However, the changes were lower than Minimal Detection Changes (MDC) which were obtained on previous study (ANT—5.87%, PM—7.84%, PL—7.55%) [56].

We found no significant changes in the YBT posterolateral (for the left and right lower limb), posteromedial, and composite score for left lower limbs between pretraining and post-training in the exercise group in the young footballers (Table 4).

## 4. Discussion

The aim of the study was to evaluate the effect of a three-exercise program on knee kinematics and dynamic lower limb balance in young soccer players who demonstrate dynamic knee valgus.

There were statistically significant changes (*p* < 0.05) in the YBT scores. The right lower limb reach distance for the ANT, PM, and COM directions after the exercise program increased (4.5%, 5.7%, 5.7%, respectively) and for the left limb for ANT direction (3.6%) in the exercise group at post-training. However, the changes were not considered as true change and clinically meaningful change because this change was lower than the Minimal Detection Change (MDC) which were obtained in a previous study (ANT—5.87%, PM—7.84%) [56]. According to the available literature from scientific databases, there is no MDC data for composite at YBT. The tendency to increase the dynamic balance among the participants could result from the specificity of the exercise to strengthen the popliteus muscle, which requires the standing position of one leg with simultaneous movement of the free lower limb with band resistance. Furthermore, the differences between the intervention and the control group during the 6-week period between the stages were not significant. This result rejects the hypothesis that the intervention group will obtain higher values than the control group among football players with knee valgus displacement.

Referring to publications investigating the impact of interventions on dynamic balance, most researchers focused on neuromuscular training such as torso stabilization exercises and complex strength exercises such as the squat. Filipa et al., 2012 showed significant improvement in the composite direction for the right and left limbs in young footballers after an 8-week program [57]. Imai et al. 2014 showed that a 12-week program of torso stabilization exercises has a positive effect on posterior lateral and posterior medial directions in footballers [58]. Both research groups used the Star Excursion Balance Test (SEBT) for measuring dynamic balance, they also differed in intervention, study population and anthropometric characteristics.

A primary objective of the current study was to investigate the effect of three exercises on improving knee kinetics in young athletes demonstrating dynamic knee valgus. The results showed that our 6-week strengthening exercise program was not effective in reducing knee valgus angles.

Our findings are consistent with that of Palmer et al., 2015, in which the effect of individual hip exercises on knee kinematics as assessed by single leg squat and single leg landing tests in forty-two military personnel. It is worth noted that the hip abduction strengthening exercise was the same as in our study, but differed in external resistance and intensity (everyday exercises). The results of this study showed a trend to reduce knee valgus by 5°, however, the changes were not significant [59].

Previous studies examine the effectiveness of exercises in reducing dynamic knee valgus consisted mainly of multiple exercises [29,30,31,33], using specialized equipment [32], requiring longer time [30] and biofeedback [29]. Our study demonstrates value to these studies, showing that 6-week exercises for the hip (gluteus medius), knee (popliteus), and ankle (posterior tibialis) are not effective in reducing dynamic knee valgus and increasing dynamic balance in the population of young soccer players with observed knee valgus. Despite the tendency to reduce the baseline valgus angles to post-training for the left knee by −2.62° in the exercise group, this difference was both statistically insignificant (*p* > 0.05) and clinically insignificant (<4°) [24]. Moreover, there were no differences in scores between groups (*p* > 0.05).

In the present study, we focused on athletes who demonstrate dynamic knee valgus because exercise programs are most beneficial for people who exhibit poor knee control as a risk of future injury [34]. We set the valgus criterion at ≤15° following the study by Räisänen et al. 2015, which assigned “poor knee control” for participants with FPPA valgus angle at 18.1° for non-dominant leg and 18.7° for the dominant leg in SLS test [44].

The failure of the intervention for DKV results and dynamic balance is likely to be due to the fact that the program was too short a period of time, and the lack of complex exercises such as squat, also using biofeedback.

This work should be considered in light of restrictions. There are restrictions on the use of 2D analysis to evaluate limb movement. First of all, low sensitivity in measuring changes in small angles. The results were not verified with the gold standard for evaluation of lower limb kinematics, which is 3D analysis. However, the angles of the extremities in the frontal projection in 2D are correlated with 3D results [43]. Kinematics assessment using Kinovea software used in this paper seems to be simpler to reproduce and without exposing costs in clinical settings. In addition, it requires no experience in video analysis and is easy to use to obtain accurate and reliable kinematic values of motion patterns [48]. Moreover, due to the lack of access to specialized equipment, muscle strength assessment was not used to investigate whether the strengthening training increased the force generation capacity of gluteus medius, popliteus, and tibialis posterior muscles. The limitations of the current study include the small sample size. We did not perform a priori power analysis in our study and it was probably underpowered. It is worth mentioning that in the current study there was a large dropout, in the exercise group—five participants, in the control group—fiveparticipants, which accounts for 26% loss of participants, including three who players sustained lower limb injuries during training or a football match. The results of the current study reject the hypothesis that the three strengthening exercises had an effect on changes in kinematics and balance in young soccer players.

## 5. Conclusions

This study demonstrated that there were no statistically significant differences between groups in dynamic knee valgus angles and dynamic balance values after 6 weeks of strengthening exercises of the gluteus, popliteus and posterior tibialis muscles in young footballers with poor knee control. Despite statistically significant changes in the exercise group for the lower right limb for ANT, PM, and COM, and the left limb for ANT, these changes are clinically insignificant. Future research, taking into account our results, should focus on more comprehensive exercises, where possible using biofeedback methods to improve knee kinematics.

## Figures and Tables

**Table 1:** Exercises description.

Exercise	Starting Position	Exercise	Week 1	Week 2–3	Week 4–6
Gluteus medius [39]	Side-lying, free leg flexed 90° in the knee joint, exercised leg straightened. The hand on the side of the exercised leg lies on the hip. Band tied over the patella.	Hip abduction to an angle of about 30° in the hip joint.	2 sets 10 reps	3 sets 10 reps	3 sets 15 reps
Popliteus [41]	Standing on one leg. Hands resting on the hips. Band applied to the forefoot.	Flexion movement in the knee joint with simultaneous external rotation of the thigh and internal rotation of the tibia.			
Tibialis posterior [40]	Sitting position with knees flexed approximately at 90°, the forearm on the side of the exercised leg based on the knee. The heel is in contact with the ground. Band hooked at the forefoot.	Resisted foot adduction and internal rotation of the tibia without femur movement.			

**Table 2:** Anthropometric characteristics of participants.

Characteristics	Exercise Group (*n* = 22)	Control Group (*n* = 23)	*p*-Value
Age, median (IQR), years	13 (12–15)	13 (12–15)	0.847 ^†^
Height, mean (SD) centimeters	157 (10)	158 (14)	0.877 ^‡^
Weight, mean (SD) kilograms	45 (10)	45 (12)	0.943 ^‡^
BMI, median (IQR) kg/m^2^	17 (16–19)	18 (16–19)	0.883 ^†^
Skeletal muscle mass, median (IQR) kilograms	19 (17–23)	18 (17–28)	0.892 ^†^

IQR—Interquartile range, SD—Standard Deviation, BMI—Body Mass Index, ^†^—Mann-Whitney U test, ^‡^—independent *t*-test.

**Table 3:** Comparison within-group and between group for the dynamic knee valgus.

Physical Performance Measure (°)		Exercise	Control	Between Groups	*p*-Value	d Cohen’s
Dynamic valgus left—mean, (SD)	Pre	16.07 (8.75)	18.03 (7.53)	1.95	0.426	0.24
	Post	13.45 (6.60)	18.70 (11.36)	5.25	0.108	0.57
					
	Post-Pre	−2.63	0.68			
Dynamic valgus right—mean, (SD)	Pre	16.33 (10.28)	18.48 (9.41)	2.14	0.469	0.22
	Post	16.96 (11.12)	19.84 (9.73)	2.88	0.427	0.27
	Post-Pre	0.63	1.37			

**Table 4:** Comparison within-group and between group for the Y-Balance Test.

Y-Balance Test (LL%)	Exercise Group		Within-Group *p*-Value	Control Group		Within-Group *p*-Value	Between-Groups *p*-Value	d Cohen’s
	Pre	Post		Pre	Post		Post	
ANTERIOR right—median, (IQR)	69.1 (8.1)	73.6 (8.3)	0.038 *	70.5 (7.0)	70.3 (9.1)	0.687	0.215	0.44
ANTERIOR left—median, (IQR)	68.3 (7.2)	71.9 (7.3)	0.039 *	72.3 (5.5)	72.1 (7.7)	0.831	0.757	0.11
POSTEROLATERAL right—median, (IQR)	106.3 (7.8)	112.9 (4.7)	0.055	105.6 (8.0)	107.5 (8.2)	0.381	0.054	0.71
POSTEROLATERAL left—mean (SD)	108.3 (8.8)	111.6 (6.8)	0.208	108.0 (8.1)	109.3 (6.6)	0.583	0.286	0.34
POSTEROMEDIAL right—median (IQR)	104.3 (12.3)	110.0 (11.3)	0.006 *	105.5 (11.1)	108.01 (7.9)	0.831	0.352	0.33
POSTEROMEDIAL left—mean (SD)	105.0 (9.0)	107.2 (6.2)	0.394	108.0 (11.4)	107.8 (6.5)	0.959	0.654	0.09
COMPOSITE RIGHT—median (IQR)	92.2 (5.9)	97.9 (6.3)	0.015 *	93.8 (7.3)	95.1 (5.3)	0.355	0.113	0.57
COMPOSITE left—mean (SD)	94.4 (7.19)	96.8 (5.3)	0.257	95.9 (7.0)	96.0 (5.6)	0.966	0.605	0.15

IQR—interquartile range, SD—Standard Deviation, for non-parametric data Wilcoxon and Whintey–Mann tests were used, for parametric test data *T* tests were used, * Statistically significant (*p* < 0.05).

## Data Availability

No new data were created or analyzed in this study. Data sharing is not applicable to this article.
